# The miRNA-mRNA Regulatory Modules of *Pinus massoniana* Lamb. in Response to Drought Stress

**DOI:** 10.3390/ijms241914655

**Published:** 2023-09-28

**Authors:** Xinhua Chen, Hu Chen, Tengfei Shen, Qunfeng Luo, Meng Xu, Zhangqi Yang

**Affiliations:** 1 Research Institute of Tropical Forestry, Chinese Academy of Forestry, 682 Guangshan Road 1, Guangzhou 510520, China; xinhua_chen108@163.com; 2 Co-Innovation Center for Sustainable Forestry in Southern China, Key Laboratory of Forest Genetics and Biotechnology Ministry of Education, College of Forestry, Nanjing Forestry University, 159 Longpan Road, Nanjing 210037, China; stf.njfu@outlook.com; 3 Engineering Research Center of Masson Pine of State Forestry Administration, Engineering Research Center of Masson Pine of Guangxi, Guangxi Key Laboratory of Superior Timber Trees Resource Cultivation, Guangxi Forestry Research Institute, 23 Yongwu Road, Nanning 530002, China; chenhubeijing-2008@163.com (H.C.); lqf20060388@163.com (Q.L.)

**Keywords:** masson pine, sRNA-seq, degradome, microRNA, drought, root

## Abstract

Masson pine (*Pinus massoniana* Lamb.) is a major fast-growing woody tree species and pioneer species for afforestation in barren sites in southern China. However, the regulatory mechanism of gene expression in *P. massoniana* under drought remains unclear. To uncover candidate microRNAs, their expression profiles, and microRNA-mRNA interactions, small RNA-seq was used to investigate the transcriptome from seedling roots under drought and rewatering in *P. massoniana*. A total of 421 plant microRNAs were identified. Pairwise differential expression analysis between treatment and control groups unveiled 134, 156, and 96 differential expressed microRNAs at three stages. These constitute 248 unique microRNAs, which were subsequently categorized into six clusters based on their expression profiles. Degradome sequencing revealed that these 248 differentially expressed microRNAs targeted 2069 genes. Gene Ontology enrichment analysis suggested that these target genes were related to translational and posttranslational regulation, cell wall modification, and reactive oxygen species scavenging. miRNAs such as miR482, miR398, miR11571, miR396, miR166, miRN88, and miRN74, along with their target genes annotated as F-box/kelch-repeat protein, 60S ribosomal protein, copper-zinc superoxide dismutase, luminal-binding protein, S-adenosylmethionine synthase, and *Early Responsive to Dehydration Stress* may play critical roles in drought response. This study provides insights into microRNA responsive to drought and rewatering in Masson pine and advances the understanding of drought tolerance mechanisms in *Pinus*.

## 1. Introduction

Drought is one of the most significant natural environmental factors that affect plant growth, yield, and survival [1]. As sessile organisms, plants have evolved mature mechanisms to cope with drought, for example, stomatal regulation, protective solute accumulation, reactive oxygen species (ROS) detoxification, and cell wall stiffening [2]. Woody tree plants are often challenged by drought stress during their long lifespan [3]. Many large-scale forest mortality events caused by drought have been documented [4,5]. Moreover, the ongoing global climate change is making drought events more frequent, longer-lasting, and more intense [6,7,8], resulting in severer loss. Therefore, investigation into the underlying mechanism of how woody tree plants respond to drought will help to improve their drought tolerance and maintain growth and productivity [9,10].

Roots play an important role in plant responses to drought. They are responsible for water uptake in the whole plant and are also the first organ to sense soil-borne water deficit [11,12]. Roots respond to drought by a variety of mechanisms at multiple levels, including morphological and anatomical, physiological, and biochemical levels [13,14,15]. These changes are underpinned by molecular responses, such as the regulation of gene expression [16,17]. For instance, root-specific overexpression of *OsERF71* resulted in larger aerenchyma, more cell layers in the root vasculature, and significantly increased drought tolerance in rice [18]. Overexpression of *PuC3H35* reduced hydrogen peroxide and superoxide anion content in roots and increased drought tolerance in *Populus ussuriensis* [19]. Overexpression of the *84KHDA909* from 84K poplar increased root growth and drought tolerance in *Arabidopsis* [20]. Overexpression of *PdNF*-*YB7* from *Populus* increased *Arabidopsis* primary root length and enhanced water use efficiency and drought tolerance [21]. Regulation of the expression of these genes involves multiple mechanisms, including microRNA-mediated expression regulation [22].

microRNAs (miRNAs) are endogenously expressed non-coding small RNAs, of typically 20–24 nt, which act as post-transcriptional regulators of gene expression through sequence complementarity [23,24]. Plant miRNAs primarily function through two mechanisms: transcript cleavage and translation repression [24,25,26]. miRNAs are versatile regulators in plant development, growth, and response to abiotic stress [25,27]. They extensively regulate plant responses to drought stress [22,28]. Recent studies have identified additional miRNAs critical for plant drought responses [29,30,31]. sRNA-seq technology is a powerful tool for discovering drought-related miRNAs [32]. It has been extensively applied in miRNA studies on drought response in plants [33,34,35]. *Pinus* is a diverse genus of trees that has a wide distribution throughout the Northern Hemisphere [36]. However, to date, the study of *Pinus* on the response of miRNAs to drought has only been reported in the Mediterranean pine species *Pinus pinaster* [37]. The response of miRNAs to drought in other pine species from different habitats has not been reported.

Masson pine (*Pinus massoniana* Lamb.) is one of the main coniferous trees in southern China, with a wide distribution between 21°41′ to 33°56′ N and 102°10′ to 123°14′ E, and an elevation range of 600 to 1650 m [38]. Masson pine has the advantages of being broadly adaptive, fast-growing, and having high-quality wood, making it one of the main afforestation trees in southern China [39]. The total area of forests with Masson pine as the major species sums up to 80.43 (including plantations of 2.52) million hectares in China [40]. In addition, Masson pine shows remarkable drought tolerance [39]. To date, sRNA-seq has been utilized in Masson pine to identify candidate miRNAs that may be involved in response to nematodes [41], low-phosphorus stress [42,43], and in strobilus development [44] and in xylem development [45]. However, no studies have been reported on the miRNA response to drought in Masson pine. We hypothesize that the expression levels of miRNA from multiple families respond to drought and/or rehydration, and they are involved in regulating the drought adaptation of Masson pine roots through targeting various mRNAs. Thus, we constructed 21 sRNA-seq libraries from Masson pine roots of seedlings treated by drought and rehydration to identify miRNAs and evaluate their expressions. Moreover, the target genes of the miRNAs were identified by degradome sequencing. In addition, ten miRNA genes were selected for qRT-PCR verification. Our study aims to identify drought responsive miRNA, clarify the expression profile of key miRNAs involved in drought response, and provide information about the miRNA-mediated regulatory network of gene expression under drought in Masson pine.

## 2. Results

### 2.1. Identification of Known and Novel miRNAs

**Figure 1:**
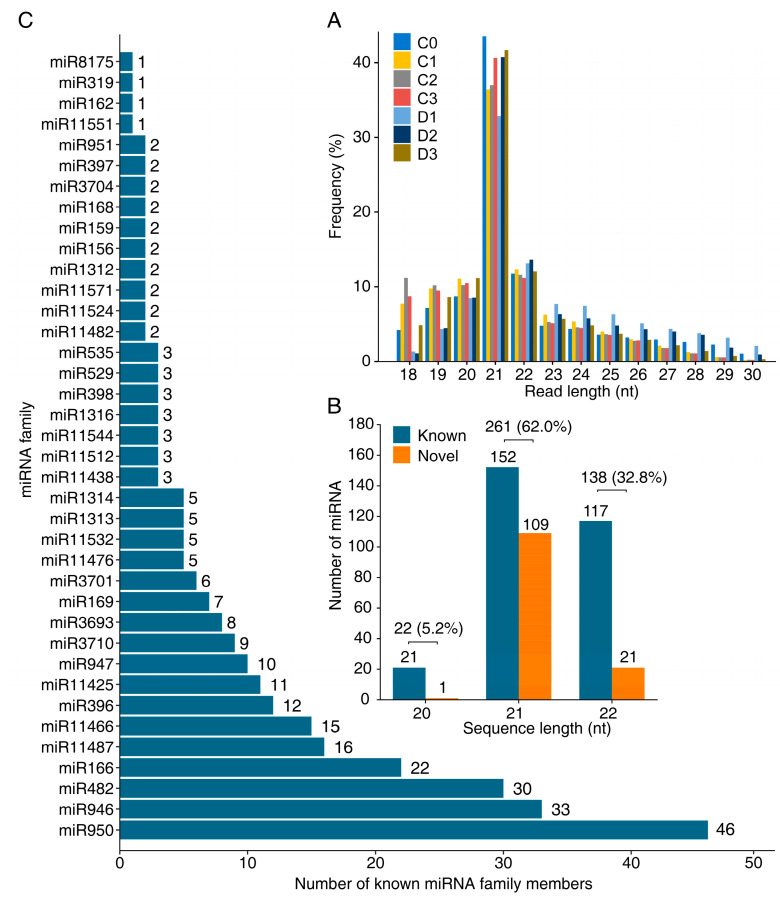
Size distribution of and member counts of miRNA family. (**A**) Histogram for read length frequency for 18–30 nt clean reads in sRNA libraries. (**B**) Histogram for counts of identified 421 miRNAs at different lengths. (**C**) Histogram for counts of members within each miRNA family.

In the context of the 21 libraries, following the exclusion of reads below 18 nt and above 30 nt, the sRNA clean reads predominantly ranged from 20 to 22 nt, with particular prevalence seen in 21 nt reads (Figure 1A). Then, to identify the miRNAs, these clean reads were aligned to the *P. massoniana* transcriptome from our previous study on the same set of samples [46]. The mapping rates ranged from 63.71% to 78.93% across the 21 libraries (Table S1). A total of 421 miRNAs were identified, mainly at 21 nt, followed by 22 nt and 20 nt, comprising 261 (62.00%), 138 (32.8%), and 22 (5.2%) counts, respectively (Figure 1B). Among them, 290 and 131 were known and novel miRNAs, respectively. Within the 290 known miRNAs, 21, 152, and 117 were 20 nt, 21 nt, and 22 nt long, respectively. These 290 known miRNAs belonged to 38 miRNA families, such as miR950 (46 members), miR946 (33 members), and miR482 (30 members, Figure 1C). Among the 131 novel miRNAs, 1, 109, and 21 were 20 nt, 21 nt, and 22 nt long, respectively. These 131 novel miRNAs represented 60 families, for instance, miRN17 (nine members), miRN54 (eight members), and miRN11 (seven members, Table S2). The nucleotide composition of the mature miRNA sequence was evaluated. Notably, these miRNAs exhibited a preference for a 5′-uridine residue (Figure S1).

### 2.2. Differentially Expressed miRNAs under Drought and Rehydration

**Figure 2:**
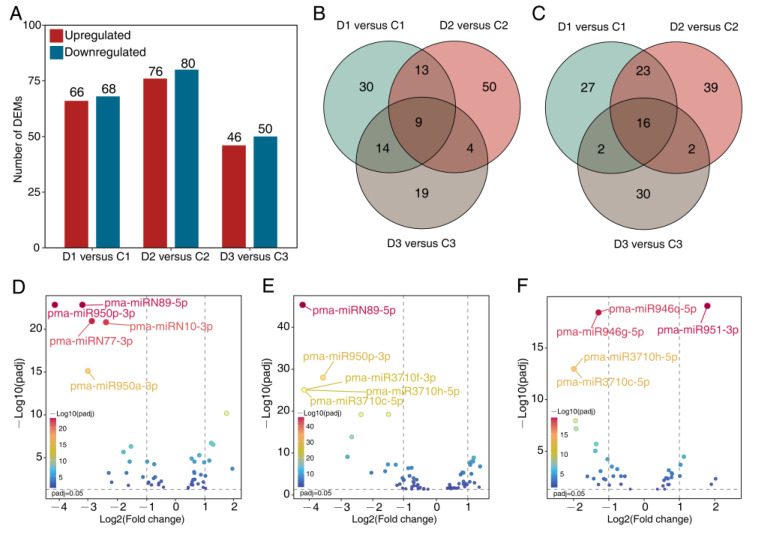
Analysis of differentially expressed miRNAs (DEMs) of the three comparisons. (**A**) Histogram for pairwise (treatment versus control) comparisons of DEM counts. Venn diagrams for comparison of upregulated (**B**) and downregulated (**C**) DEMs among and between the three pairwise comparisons. Volcano plots for DEMs in pairwise comparisons, D1 versus C1 (**D**), D2 versus C2, (**E**) and D3 versus C3 (**F**).

A total of 134, 156, and 96 differentially expressed miRNAs (DEMs) were determined in D1 versus C1, D2 versus C2, and D3 versus C3, respectively (Table S3). Among them, 66, 76, and 46 DEMs were upregulated in D1 versus C1, D2 versus C2, and D3 versus C3, respectively (Figure 2A). Furthermore, 68, 80, and 50 DEMs were downregulated in D1 versus C1, D2 versus C2, and D3 versus C3, respectively (Figure 2A). Among the upregulated DEMs, 30, 50, and 19 were uniquely differentially expressed in D1 versus C1, D2 versus C2, and D3 versus C3, respectively (Figure 2B). Among the downregulated DEMs, 27, 39, and 30 were uniquely differentially expressed in D1 versus C1, D2 versus C2, and D3 versus C3, respectively (Figure 2C). There were DEMs that were highly responsive to drought and rehydration, for example, the known miRNAs, such as pma-miR950p-3p (−4.15 fold), pma-miR950a-3p (−3.02 fold) in D1 versus C1; pma-miR950p-3p (−3.57 fold), pma-miR3710f-3p (−4.16 fold), pma-miR3710h-5p (−4.16 fold), and pma-miR3710c-5p (−4.16 fold) in D2 versus C2; pma-miR951-3p (1.79 fold), pma-miR946g-5p, pma-miR946q-5p (−1.29 fold), pma-miR3710c-5p (−1.99 fold), and pma-miR3710h-5p (−1.99 fold) in D3 versus C3; as well as the novel miRNAs, such as pma-miRN89-5p (−3.20 fold), pma-miRN77-3p (−2.67 fold), and pma-miRN10-3p (−2.39 fold) in D1 versus C1; and pma-miRN89-5p (−4.21 fold) in D2 versus C2 (Figure 2D–F, Table S3). Altogether, there were 248 unique DEMs, which were differentially expressed in at least one comparison (Table S4).

### 2.3. Expression Profile of DEMs

**Figure 3:**
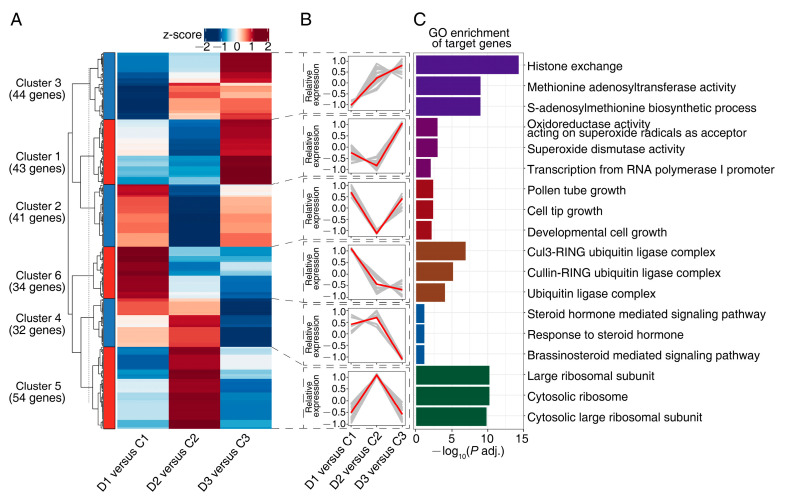
*K*-means clustering of 248 unique differentially expressed miRNAs (DEMs) and functional analysis of the target genes. (**A**) Heatmap of the DEMs grouped into six clusters with *K*-means algorithm. Red and blue colors represent upregulated and downregulated DEMs, respectively. (**B**) Expression patterns for the six clusters of DEMs. The red line indicates median values of relative gene expression. (**C**) The enriched gene ontology (GO) terms of genes targeted by miRNAs within a certain cluster. Three GO terms with the largest −log10(*p* adjusted) were shown.

Gene expression profiles can help to predict gene function [47]. Therefore, the DEMs were categorized into six clusters using *K*-means clustering analysis according to expression profiles (Figure 3A, Table S4). Cluster 1 and cluster 2 contained DEMs with peak expressions in D1 versus C1 and D3 versus C3 (drought conditions, Figure 3A,B, Table S4). Cluster 1 primarily featured miRNAs from miR946 and miR11425 families and cluster 2 exhibited a prevalence of miRNAs from miR3710 and miRN11 families. In Cluster 3, DEMs peaked in expression during D3 versus C3 (drought condition) and they were mainly from miR950 and miRN9 families. Clusters 4 and 5 contained DEMs whose expressions were peak in D2 versus C2 (post-rewatering). In cluster 4, DEMs were predominately from miR166 and miR11487 families (Figure 3C, Table S4). In cluster 5, the majority of DEMs were from miRN17 and miR11487 families. In cluster 6, DEMs culminated expression in D1 versus C1 (drought condition). The majority of DEMs in this cluster belonged to miR482 and miRN54 families.

### 2.4. Target Gene Prediction via Degradome Sequencing

Globally, 16,705,004 sequences were obtained from degradome sequencing, and 16,697,634 clean reads sequences were obtained after fastp quality control. Q20 and Q30 sequences accounted for 98.4% and 43.8% of the clean read sequences, respectively (Table S5). Within the set of 421 miRNAs, there were 419 that targeted 3582 mRNA, forming 15,522 miRNA-mRNA modules (Table S6). Of these, 2942 modules were ‘category 0’, 1626 modules were ‘category 1’, and 6995 modules were ‘category 2’. In the subset of 248 DEMs, 247 targeted 2069 genes, of which 1772 were annotated by SwissProt (Table S7). Out of 1772 annotated genes, 155 isoforms were annotated as transcription factor genes. The top five transcription factors with the highest number of genes were squamosa promoter-binding-like protein (SPL, 28 genes), dehydrin (26 genes), growth-regulating factor (23 genes), homeobox-leucine zipper protein (15 genes), transcription factor GAMYB (15 genes), and NAC domain-containing protein (nine genes). On the other hand, 1617 isoforms were annotated as functional protein genes. The top five categories of functional protein genes, ranked by the number of genes, were disease resistance protein (245 genes), TMV resistance protein (103 genes), probable disease resistance protein (60 genes), 60S ribosomal protein (29 genes), and 40S ribosomal protein (19 genes).

Further, GO term enrichment analysis was performed on DEM target genes in each cluster to infer the mediatory role of DEMs during drought and rehydration. DEMs in cluster 1 were linked to GO terms such as “oxidoreductase activity using superoxide radicals as acceptor” and “superoxide dismutase activity” (Figure 3C, Table S8). The DEMs in cluster 2 were associated with “pollen tube growth”, “cell tip growth”, and “developmental cell growth” (Figure 3C, Table S8). DEMs in cluster 3 were related to “histone exchange” and “methionine adenosyltransferase activity” (Figure 3C, Table S8). DEMs in cluster 5 were correlated with “large ribosomal subunit” and “cytosolic ribosome” (Figure 3B, Table S8). DEMs in cluster 6 were linked to “cul3-RING ubiquitin ligase complex” and “ubiquitin ligase complex” (Figure 3C, Table S8).

### 2.5. The Negatively Correlated miRNA-mRNA Modules

**Figure 4:**
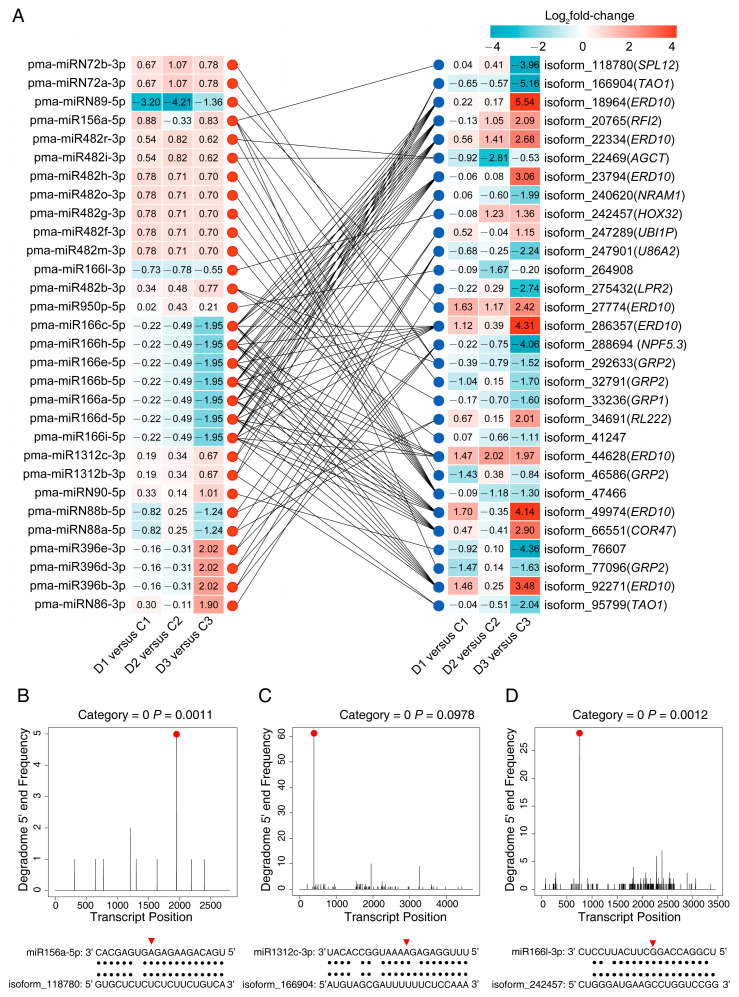
Expression profiles of important miRNA-mRNA modules and their validation. (**A**) A combined view of the expression profile of negative-correlated modules of differentially expressed microRNAs (DEMs) and differentially expressed genes (DEGs) under drought and rehydration. The heatmap on the left presents the expression of the DEMs, and the heat map on the right presents the expression of the DEGs. The red and blue dots represent DEMs and DEGs, respectively, and the solid black lines in between represent the degradome-validated targeting relationships between the DEMs and the DEGs. T-plots and miRNA-mRNA alignments represent pma-miR156a-5p cleaves isoform_118780 (**B**), pma-miR1312c-3p cleaves isoform_166904 (**C**), pma-miR166l-3p cleaves isoform_24257 (**D**). The red dots and triangles represent the cleavage positions on the target genes.

Taking advantage of the available transcriptomics data for the same samples from our previous study [46], negatively correlated DEM-target modules were identified through Pearson’s correlation analysis. This analysis involved assessing the normalized DEM expressions (average RPM of three biological replicates) and target expressions (average FPKM of three biological replicates). Given the complexity of the network, which hindered effective visualization, a concise mini-miRNA network was generated using three criteria: (i) inclusion of degradome signals with quality categories zero, one, or two at the cleavage sites of the targets; (ii) selection of differentially expressed genes (DEGs) from the transcriptome as targets; (iii) and a DEM-target expression coefficient of r < −0.80. As a result, 100 negatively correlated DEM-DEG modules were identified, containing 30 DEMs and 30 DEGs (Table S9). For a more intuitive presentation, log_2_(fold change) corresponding to these DEMs and DEGs was used to visualize the expression profiles (Figure 4A).

Within this network, eleven miRNAs exhibited multiple gene targeting. For instance, pma-miR166a-5p targeted *Early Responsive to Dehydration Stress* (*ERD10*, isoform_18964), dehydrin (*COR47*, isoform_66551), and E3 ubiquitin-protein ligase (*RFI2*, isoform_20765). Additionally, seven miRNAs were found targeting of more than one gene, such as pma-miR482b-3p which targeted glycine-rich cell wall structural protein (*GRP1*, isoform_33236) and isoform_41247 (No SwissProt annotation). Conversely, twelve miRNAs exclusively targeted single gene, exemplified by pma-miR166l-3p targeting homeobox-leucine zipper protein (*HOX32*, isoform_242457). On the other hand, thirteen target genes were targeted by more than two miRNAs. For instance, isoform_286357 (*ERD10*) was targeted by seven miRNAs, including pma-miR166b-5p, pma-miR166e-5p, and pma-miR166h-5p. Additionally, six target genes were involved in more than one miRNA interaction, such as polyubiquitin (*UBI1P*, isoform_247289) which was targeted by pma-miRN88a-5p and pma-miRN88b-5p. Furthermore, eleven genes were targeted by only one gene, exemplified by squamosa promoter-binding-like protein (*SPL12*, isoform_118780) targeted by pma-miR156a-5p.

## 3. Discussion

Studies have demonstrated that miRNAs regulate drought response in crop plant species [22,48,49] as well as forest tree species [50]. Pine trees respond to drought through a complex process that involves the expression reprogramming of multiple genes [51,52]. A study conducted on *P. pinaster* showed that a significant number of miRNAs were involved in the drought response of pine trees [37]. Roots play a crucial role in a plant’s response to drought [53,54,55]. However, the role of miRNAs in the roots of Masson pine trees in response to drought and rewatering has not been reported yet. Here, we employed high-throughput small RNA sequencing to identify miRNAs and their expression profiles from 21 libraries under control and stress conditions to study the effect of drought and rehydration in seedling root of *P. massoniana*. A total of 421 miRNAs were identified, among which 248 miRNAs exhibited differential expression under drought stress and rewatering. Through degradome sequencing, potential miRNA-mRNA regulatory modules were predicted.

### 3.1. Features of P. massoniana miRNA Population

In this study, each library generated a minimum of 19,969,315 clean reads, providing sufficient sequencing depth for subsequent analysis. The reads exhibited high quality, with Q20 base ratio > 99.50%, and the Q30 base ratio was >92.51%. The most abundant read lengths were 21 nt, followed by 20 nt and 22 nt, consistent with previous finding in *P. massoniana* [40,43] and *Pinus tabuliformis* [56]. Here, the 421 miRNAs were 20, 21, and 22 nt long, which is a characteristic commonly found in plant miRNAs [57]. Sequences of 21 and 22 nt were the most prevalent in both known and novel miRNAs (Figure 1B). Moreover, the mature miRNA had a strong bias toward a 5′-uridine residue (Figure S1), consistent with previous observation in pine [44,52]. These factors, namely the length and the preference of 5’-uridine residue, can influence the partitioning of miRNAs onto specific AGO proteins [58,59,60], thereby affecting the functions performed by these AGO proteins [57]. AGO1 is considered the most essential protein in the miRNA pathway [61,62] and it prefers to bind 21 nt miRNAs with a 5’-uridine residue [57,58].

### 3.2. Stress Responsive miRNAs Families in P. massoniana Root

A total of 248 miRNAs in *P. massoniana* roots were differentially expressed under drought and rewatering, representing diverse families of plant miRNAs (Table S3). Some of these miRNAs have been previously reported. For instance, miR529 and miR156 family members were upregulated under drought stress (D1 and D3, Table S4), similar to their counterparts in rice [63] and maize [64], respectively. Conversely, miR159, miR166, miR168 and miR398 members were downregulated under drought stress (D1 and D3, Figure 4A, Table S4), consistent with findings in alfalfa [65], rice [66], *Ammopiptanthus mongolicus* [67], and pea [68], respectively. Interestingly, the miR397 and miR535 families showed contrasting expression patterns at different stages of drought stress (D1 and D3, Table S4), indicating their distinct regulatory roles. In addition, the miR396, miR482 and miR950 displayed multiple expression patterns during drought stress (D1 and D3, Table S4). Noticeably, four members of the miR482 family were downregulated, while the remaining nine members were upregulated. These observations suggest that members within the same miRNA family may regulate target genes differently. Moreover, 17 *Pinaceae*-specific miRNA families were identified: miR946, miR947, miR951, miR1312, miR1313, miR3693, miR3701, miR3704, miR3710, miR11425, miR11466, miR11476, miR11482, miR11487, miR11512, miR11524, and miR11571 (Table S4). Among these, miR946, miR947, miR951, miR1313, miR3704, and miR11425 were also found in *P. pinaster* roots under drought stress [52]. Additionally, 77 DEMs belong to 35 novel miRNA families, implying the existence of a specific regulatory mechanism in *P. massoniana*.

### 3.3. miRNA Modules Mediate Translational Regulation in Drought Response

Ribosomal proteins (RPs) are components of ribosomes and perform multiple roles in biological processes, such as ribosome biogenesis, protein synthesis, cell growth, development, and abiotic stress response [69]. For example, RP genes could be induced by water deficit in rice root [70,71] and enhance the expression of two RP genes, respectively, as both resulted in improved drought tolerance in rice [70]. Moreover, knockdown of *60S ribosomal protein L14-2* resulted in reduced tolerance to drought stress in cotton [72]. Here, DEMs from five miRNA families: miR482, miR11524, miRN88, miR11476, and miR396 were found to target fifteen isoforms annotated as ribosomal protein (Table S8). Notably, the expression of pma-miRN88a-5p and pma-miRN88b-5p were negatively correlated with their target gene, isoform_34691 (*RL222*), by drought in D1 versus C1 and D3 versus C3 (Figure 4A). These findings suggest that these miRNAs may regulate the ribosome and play a role in the drought response of *P. massoniana*.

Environmental stress activates unfolded protein response (UPR) in the endoplasmic reticulum (ER), a highly conserved response in plants [73,74]. Persistent UPR can cause programmed cell death, so UPR is under tight control [75,76]. ER-resident luminal-binding protein (BiP), a central UPR regulator [77,78], aids protein folding, re-establishing ER homeostasis [79,80,81]. *BiP* plays a vital role in drought tolerance. Overexpressing *BiP* improved drought tolerance in plants [82,83,84,85]. However, overproduced BiP proteins suppressed the expression of *BiP*, indicating a negative feedback mechanism of the UPR, whereby the cell may reduce nonessential *BiP* transcripts when functional BiP proteins are sufficient in protein folding during ER stress [86]. Similar observations have been made in yeast and mammalian cells, where the overexpression of functional BiP protein mitigates UPR [87,88]. In our study, pma-miR11571-5p was found to target four *BiP* genes and was upregulated by drought stress in D1 versus C1 and D3 versus C3 (Tables S4 and S8). The results indicate that pma-miR11571-5p was involved in the negative feedback regulation by suppressing the *BiP*’s expression, thus maintaining the homeostasis of ER under drought stress.

Rapid responses to environment perturbation are vital for plants due to their sessile lifestyle [89]. Such responses, like signal transduction and cell cycle control, require prompt elimination of certain proteins, such as misfolded proteins or various normal short-lived regulators [89,90]. The ubiquitin proteasome system (UPS) mediates a major pathway responsible for protein degradation [91]. The UPS is initiated with a conserved cascade reaction involving E1, E2, and E3 enzymes, leading to the attachment of ubiquitin to specific proteins [89]. The most common E3 ligase in plants is the Skp1-Cullin-F-box (SCF) protein complex, which recognizes specific substrates through the binding interaction between SKP1-like ASK and F-box proteins [92,93,94]. Among the F-box protein family, F-box kelch proteins (FBKs) represent one of the largest subfamilies [93,95]. Previous studies in *Arabidopsis* [96] and wheat [97,98,99] have demonstrated that FBKs binds to ASK proteins and that the overexpression of *FBK* enhanced drought tolerance in plants. In our study, miR482 family members were found to target, with high confidence (category = 0), six genes annotated as *FBK* (Table S8). miR482 has also been shown to target F-box genes in lychee [100] and strawberry [101]. Therefore, our findings suggest that members of miR482 family played a role in drought response in *P. massoniana*.

### 3.4. miRNA Modules Mediate Cell Wall Modification in Drought Response

S-adenosylmethionine synthase (SAMS) catalyzes the synthesis of S-adenosylmethionine (SAM) from methionine and adenosine triphosphate (ATP) [102]. SAM is involved in multiple transmethylation reactions, including those related to lignin biosynthesis [103,104]. Increasing deposition of lignin in cell walls may be one of the mechanisms by which cells respond to drought [105,106,107]. Methylation of lignin precursors is a critical step in lignin synthesis, with SAM acting as the primary methyl group donor [108,109]. Previous studies on *Pinus banksiana* [110], peanut [111], soybean [112], and cucumber [113] have shown that the expression levels of SAMS protein and/or transcript in roots were responsive to drought stress. In this study, members from miR396 and miRN74 were found to target eight isoforms annotated as SAMS (Table S8). These genes were enriched in two GO pathways, “methionine adenosyltransferase activity” and “S-adenosylmethionine biosynthetic process” (Figure 3D, Table S8). Their findings suggest that miR396 and miRN74 was involved in lignin biosynthesis during drought in *P. massoniana* by mediating the expression of *SAMS.*

*HOX32* encodes a transcription factor that belongs to the HD-ZIP III group [114]. In our study, isoform_242457, annotated as *HOX32*, was found to be targeted by pma-miR166l-3p (Figure 4A, Table S9). A previous study on rice has demonstrated that miR166 targets *OsHOX32*, and knockdown of miR166, or overexpression of *OsHOX32* led to a reduction in lignin content in cell wall [114]. Many studies have shown that drought increased lignin accumulation in the roots [105,106,107]. However, since lignin biosynthesis consumes a high and irreversible input of carbon sources, its deposition is tightly regulated through transcriptional, posttranscriptional, and posttranslational processes [114,115,116]. In our study, the pma-miR166l-3p were downregulated, while the expression of its target gene *HOX32* were upregulated in D2 versus C2 (rewatering) and D3 versus C3 (drought) stages. These results suggest that the pma-miR166l-3p:*HOX32* module may be involved in fine-tuning of lignin biosynthesis under drought and rehydration. *HOX32* was slightly downregulated in D1 versus C1 (drought). This may be due to other factors regulating its expression [117].

### 3.5. miRNA Modules Mediate ROS Scavenging in Drought Response

ROS are free radicals of oxygen. They may have both beneficial and harmful effects [118]. During drought, ROS can accumulate excessively in cells, leading to oxidative stress [119]. To counteract this, cells possess various enzymes, such as superoxide dismutase (SOD), which provide the first line of defense against oxidative stress [120]. The copper-zinc superoxide dismutase (CSD) is the most common type of SOD [121]. Previous studies have shown that miR398 regulates drought tolerance in plants by targeting *CSD* genes [122,123,124]. Further, miR398 was found to be downregulated in the roots by drought stress in pea [68] and legume [123]. Overexpression of miR398 reduced the expression of *CSD* and impaired plant drought tolerance [124,125], while the knockdown of miR398 increased *CSD* expression and enhanced plant drought tolerance [124]. In this study, pma-miR398a-3p, pma-miR398b-3p, and pma-miR398c-3p targeted three genes annotated as *CSD* (Figure 4A, Table S8). The expression of these pma-miR398 was downregulated in D1 versus C1 and D3 versus C3 (drought). These findings suggest that the downregulation of pma-miR398 make root cells to produce more SOD enzymes, enabling them to scavenge peroxides induced by drought stress.

### 3.6. Putative miRNA-Mediated Regulatory Network

Based on DEM–target correlations, a schematic model was proposed for miRNA-mediated regulatory network during drought and rehydration (Figure 4A, Table S9). In this network, pma-miR166 targeted the most genes among the negatively correlated miRNA-mRNA modules (Figure 4A). In addition to *HOX32* as previous mentioned, pma-miRNA166 members were found to target *Early Responsive to Dehydration stress* (*ERD*) genes such as *ERD10*. ERD10 is a dehydrin protein [126], which is a potent chaperon that activates other protective proteins or acts as a plasma membrane stabilizer to protect cells [127,128]. The expression of *ERD10* can be rapidly increased by dehydration [126] and *erd10* mutants show reduced drought tolerance [129]. Here, pma-miR166a-5p and six other members were downregulated, while *ERD10* was highly upregulated by drought in D1 versus C1 and D3 versus C3. This result implies that reduced pma-miR166 expression promoted ERD protein expression in response to drought. Noticeably, pma-miRN89-5p, which was substantially downregulated by drought in D1 versus C1 and D3 versus C3, (Figure 4A) targeted another *ERD10* gene. This result indicates that pma-miRN89-5p plays a role in drought response. Moreover, pma-miR156a-5p and pma-miR482b-3p targeted *GRP* genes, including cell wall-associated GRP (isoform_292633 and isoform_33236) and RNA-binding GRP (isoform_32791, isoform_46586 and isoform_77096; Figure 4A). Cell wall-associated GRPs have been reported to be involved in cell elongation [130] and root size control [131]. As for RNA-binding GRP, they may play a role in RNA stabilization, processing, and transport according to a previous report [132]. miR396 plays a pivotal role in regulating plant architecture through its mediation of gibberellin (GA) signaling [133,134]. GA signaling is subjected to regulation by hormone transporters, such as NITRATE TRANSPORTER1/PEPTIDE TRANSPORTER (NPF) [135,136]. *NPF3* overexpression dramatically inhibited root growth [135]. Moreover, the suppression of GA activity resulted in improved plant drought tolerance [137,138]. In this study, pma-miR396 were found to target one gene annotated as Protein NRT1-PTR FAMILY 5.3 and PTR4 (Table S9), which is encoded by *NPF5.3* gene (Figure 4A). And, here, *NPF5.3* was downregulated by drought treatment (Figure 4A). This result indicated that the miR396:*NPF5.3* module was involved in regulating GA signaling during drought in *P. massoniana*. pma-miR156a was found to target *SPL12* (Figure 4A). The miR156:*SPL* module exists in multiple species and was involved in drought response and growth regulation [139]. Thus, the pma-miR156a-5p:*SPL12* module may play a role in drought response in *P. massoniana*. In addition to targeting *RL222*, pma-miRN88a-5p and pma-miR88b-5p also targeted *UBI1P*, suggesting a role in regulating E1 activating enzyme in UPS. pma-miRN86-3p targeted *LPR2*. LPR was reported to mediate the response of root meristems during phosphate availability [140]. pma-miR1312b-3p and pma-miR1312c-3p were found to target two genes (isoform_ 166,904 and isoform_95799, Figure 4A), which were annotated as a target of AvrB operation (TAO1, Figure 4A, Table S9). TAO1 was reported to play a key role in signaling during the response to pathogens [141]. pma-miR1312 might be involved in drought response at the signaling level. In addition, miR482, miR950, and miRN90 targeted three genes, isoform_41247, isoform_264908, and isoform_76607, respectively, which currently have no SwissProt annotation. They may be novel genes in response to drought stress.

## 4. Materials and Methods

### 4.1. Plant Materials and Stress Treatments

The materials originated from *P*. *massoniana* seedlings of a full-sibling family (code: 19-309) derived from two high resin-yielding parents, GZ001 (female) and GZ549B (male). Approximately one month after germination, young seedlings were randomly allocated into three treatment groups and four control groups with each group being represented by three biological replicates. Subsequently, the seedlings were transplanted into non-woven pots (4 cm in diameter and 8 cm in depth) containing a mixture of coconut husk, loess soil, and peat in a ratio of 6:3:1 (*v*/*v*/*v*). During the drought experiment, all the sibling seedlings were cultivated under constant conditions of 26 °C, 16 h/8 h light/dark cycle, 60% relative humidity, and 80 μmol m^−2^ s^−1^ photon flux inside an RXZ-1000A-LED growth chamber (Ningbo Jiangnan Instrument Factory, Ningbo, Zhejiang, China). The experiment involved three treatments, namely, D1, which entailed withholding irrigation for seven days upon needle wilting; D2, which involved withholding irrigation for seven days and 17 h followed by rewatering and another seven-hour period of no irrigation; and D3, which entailed withholding irrigation for eight full days. The four controls C0, C1, C2, and C3 corresponded to the time points of treatment start, D1, D2, and D3, respectively, with regular watering every other day. To minimize the effects of circadian rhythm, sampling was conducted for half an hour at the same time each day. Approximately 0.5 cm from the root tip was collected and snap-frozen in liquid nitrogen. A total of 21 samples were obtained for small RNA sequencing, including three treatment groups and four control groups with three biological replicates each.

### 4.2. Small RNA Sequencing

RNA extraction was performed using the Invitrogen TRIzol^®^ Plus RNA Purification Kit (Thermo Fisher Scientific Inc., Waltham, MA, USA). The quality and quantity of RNA samples were evaluated using a NanoDrop 2000 spectrophotometer (Thermo Fisher Scientific Inc.) and an Agilent 2100 bioanalyzer (Agilent Technologies, Santa Clara, CA, USA). sRNA-seq libraries were constructed and sequenced at Beijing Genomics Institute (BGI, Shenzhen). Briefly, 1μg total RNA from each sample was separated using polyacrylamide gel electrophoresis (PAGE) and the 18–30 nt stripes were selected and recycled. The 3′ and 5′ adaptors were ligated to recycled RNA. The adapter-ligated RNA was reverse-transcribed and the cDNA product was amplified by PCR for 16 cycles. Amplified products were purified, and then quantified using a Qubit fluorometer (ThermoFisher Scientific, Massachusetts, USA). Subsequently, the purified products were used to produce single-strand circle DNA as the final cDNA library. A total of 21 libraries, namely, C0_1, C0_2, C0_3, C1_1, C1_2, C1_3, C2_1, C2_2, C2_3, C3_1, C3_2, C3_3, D1_1, D1_2, D1_3, D2_1, D2_2, D2_3, D3_1, D3_2, D3_3, were constructed and sequenced on a BGISEQ-500 platform (BGI, Shenzhen, Guangdong, China) using 50 bp single-end read chemistry.

### 4.3. De Novo Prediction and Annotation of miRNAs

The adaptor for sRNA sequencing was obtained using dnapi.py [142]. Cutadapt [143] was used to perform quality control (-j 0 -a adaptor -quality-base 33 -m 18 -M 30 -O 4 -discard-untrimmed -q 20 -max-n 0). Reads that contained more than one N base (also known as ambiguous base) and base with quality value less than 20 were discarded. Then, the obtained clean reads were mapped against the Rfam (v12.1) database, with one base mismatch permitted, to remove rRNA, tRNA, snRNA, and snoRNAs. Such reads with counts less than 10 were also discarded. Then, reads were analyzed by miREvo [144] and miRDeep-P2 [145] for identifying known miRNA as well as predicting novel miRNAs. Briefly, reads with more than 15 mapping sites in the reference transcriptome were filtered out. The length of the potential miRNA precursor should not have exceeded 300 nt. The range of the length of mature miRNA and miRNA* should have been 20–24 nt. miRNA/miRNA* duplexes should not have contained a large loop and should contain up to five mismatches.

### 4.4. Expression Analysis of miRNAs

Raw reads counts were generated by miRDeep-P2 and were normalized to reads per million (RPM = Number of reads mapped to a miRNA × 1,000,000/Total number of mapped reads from a given library). Differentially expressed miRNAs [DEMs, |log_2_ (foldchange)| > 1, adjusted *p* value < 0.05] were determined in R (v4.2.2) using package DESeq2 (v1.38.3) [146]. The number of upregulated and downregulated genes was visualized using the R package ggplot2 (v3.4.2) [147]. Venn diagrams were visualized using the R package VennDiagram (v1.7.3) [148] to show common and uniquely regulated DEMs among the three comparisons. *K*-means clustering of DEMs was performed in R using *Z*-scaled log_2_(fold-change), and the gene expression heatmap was visualized using the R package ComplexHeatmap (v2.14.0) [149]. GO term enrichment (adjusted *p* value < 0.05) of the target genes of DEMs was achieved using the enricher function in the R package clusterProfiler (v4.6.0) [150].

### 4.5. Degradome Library Construction and Target Gene Prediction

Equal amounts of RNA from both control (C0, C1, C2, C3) and drought (D1, D2, D3) treatments were pooled to construct one degradome library. The libraries were constructed following the method proposed by Fang et al. [151] and the sequencing was performed on the Illumina NovaSeq 6000 platform with 50 bp single-end chemistry at Genedenovo Biotechnology Co., Ltd. (Guangzhou, Guangdong, China). The degradome sequences were subjected to quality control using fastp (v 0.23.2) [152]. The resulting high-quality reads were then used to predict target genes using Cleaveland4 (parameters: -t -c 2) [153] with a full-length transcriptome (SRA accession number: PRJNA667166) as reference sequence from our previous study [45].

### 4.6. Validation of miRNA Expression via qRT-PCR

For the validation of miRNA expression obtained from sRNA-seq, the abundance of ten mature miRNAs was quantified via qRT-PCR. cDNAs of each of the 21 samples were synthesized using a Mir-X™ miRNA First Strand Synthesis Kit (TaKaRa, Dalian, China). Using the *ACT1* gene as an internal control, the cDNAs were verified by qRT-PCR with the corresponding mature sequences of the miRNAs used as forward primers (Table S10), and the universal miR 3’ primer, included in the Mir-X™ miRNA First Strand Synthesis Kit, as reverse primer. Reactions with three replicates for each of the samples were performed on ViiA™ 7 Real-Time PCR Systems (Applied Biosystems, Waltham, MA, USA). The relative expression data were calculated using 2^−ΔΔCT^ method [154]. The qRT-PCR results are shown in Figure S2.

## 5. Conclusions

In this study, 21 sRNA-seq libraries were sequenced to identify miRNAs in *P. massoniana* seedling roots under drought and rewatering conditions. A total of 421 miRNAs were identified and among them, 248 miRNAs were differentially expressed. The Gene Ontology enrichment analysis of predicted target genes of the differentially expressed miRNAs indicated their participation in drought response via various mechanisms such as translational and posttranslational regulation, cell wall modification, and ROS scavenging. miRNAs, such as miR482, miR398, miR11571, miR396, miR166, miRN88, and miRN74, along with target genes, such as those encoding F-box/kelch-repeat protein, 60S ribosomal protein, copper-zinc superoxide dismutase, luminal-binding protein, S-adenosylmethionine synthase, and *Early Responsive to Dehydration Stress*, could potentially have vital functions in responding to drought conditions. miRNA-mRNA modules, such as pma-miR396b-3p:*NPF5.3*, pma-miR156a-5p:*SPL12* and pma-miR1312b-3p:TAO1, could also play important roles in drought responses in *Pinus*. This study presents a valuable resource for further molecular investigation on complex regulatory network of gene expression and uncovering new players functioning in drought tolerance in *Pinus*.

## Data Availability

sRNA-seq and degradome sequencing data in this paper have been deposited in the Genome Sequence Archive [155] in National Genomics Data Center [156], China National Center for Bioinformation/Beijing Institute of Genomics, Chinese Academy of Sciences (GSA: CRA012245) and are publicly accessible at https://ngdc.cncb.ac.cn/gsa (accessed on 15 August 2023).

## Supplementary Materials

The supporting information can be downloaded at: https://www.mdpi.com/article/10.3390/ijms241914655/s1.
