# Supplementary material for: The miRNA-mRNA Regulatory Modules of Pinus massoniana Lamb. in Response to Drought Stress
Source: Int J Mol Sci. 2023 Sep 28;24(19):14655. doi: 10.3390/ijms241914655 (PMC10572226; doi:10.3390/ijms241914655)
Supplement: Supplementary file 1 [file ijms-24-14655-s001.zip › Supplementary data Figure S1-S2.pdf]

Supplementary Data

The miRNA-mRNA regulatory modules of *Pinus massoniana* Lamb. in response to drought stress

Xinhua Chen[1,2,3,†], Hu Chen[3,†], Tengfei Shen[2], Qunfeng Luo[3], Meng Xu[2,*], Zhangqi Yang[3,*]

[1] Research Institute of Tropical Forestry, Chinese Academy of Forestry, 682 Guangshan Road 1, Guangzhou 510520, China;

[2] College of Forestry & Co-Innovation Center for Sustainable Forestry in Southern China & Key Laboratory of Forest Genetics and Biotechnology Ministry of Education, Nanjing Forestry University, 159 Longpan Road, Nanjing 210037, China;

[3] Engineering Research Center of Masson Pine of State Forestry Administration & Engineering Research Center of Masson Pine of Guangxi & Guangxi Key Laboratory of Superior Timber Trees Resource Cultivation, Guangxi Forestry Research Institute, 23 Yongwu Road, Nanning 530002, China;

[*] Correspondence : xum@njfu.edu.cn (M.X.); yangzhangqi@163.com (Z.Y.)

[†]These authors contributed equally to this work.

Supplementary Figure S1−S2

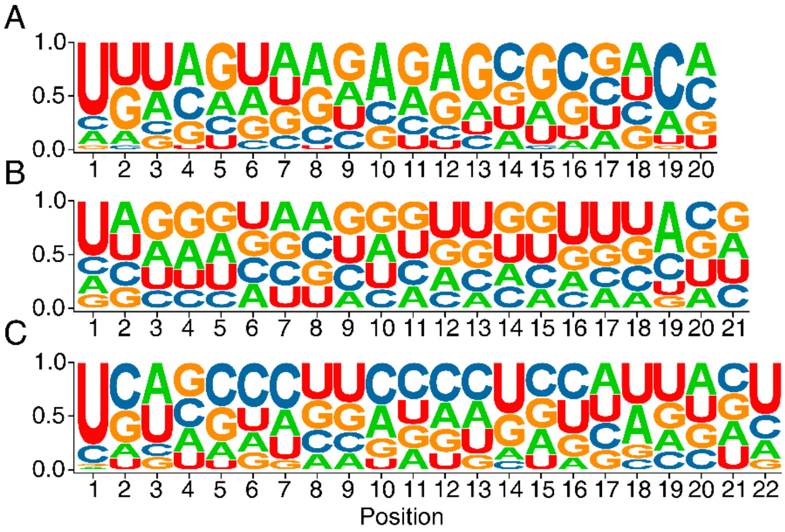

Figure S1. Base ratio at each position on a mature microRNA sequence of 20 nt (A), 21 nt (B) and 22 nt (C) long. Four letters, A, C, G, and U, represent four types of bases: adenine, cytosine, guanine, and uridine, respectively. The height of a letter indicates the ratio of a type of base at certain position on a mature microRNA sequence.

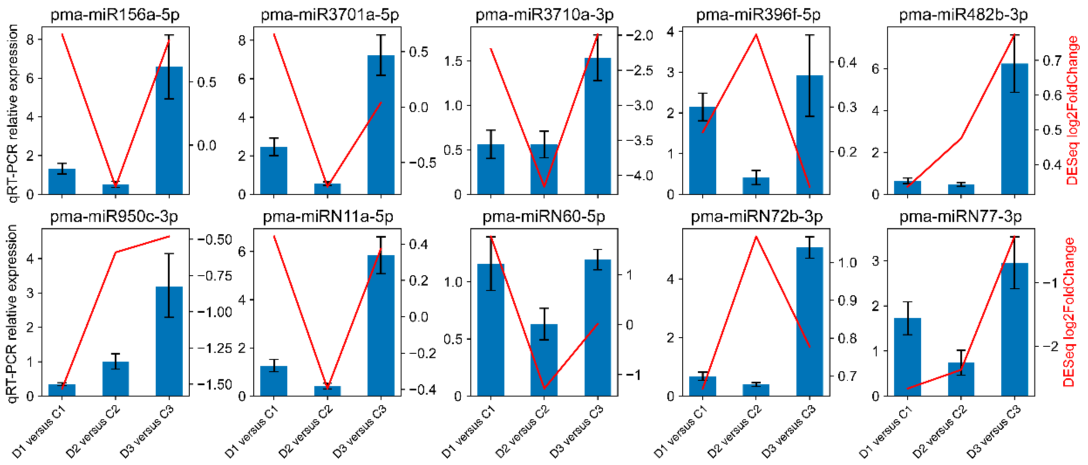

Figure S2. Quantitative real-time PCR (qRT-PCR) validation of selected miRNAs expression profiles between treatments and controls. The bar represents the relative expression level determined by qRT-PCR (left y-axis). The error bar indicates the standard error from three biological replicates. The red line represents the relative expression level of sRNA-seq (right y-axis).
